# The cerebroplacental ratio: association with maternal hypertension and proteinuria

**DOI:** 10.1590/0100-3984.2021.0026

**Published:** 2021

**Authors:** Oluwatoyin Ige Oyekale, Temitope Olugbenga Bello, Oluwagbemiga Ayoola, Adeola Afolabi, Olayemi Atinuke Alagbe, Oluwalana Timothy Oyekale, Oluwatoyin Nike Akinyoade

**Affiliations:** 1 Federal Teaching Hospital Ido Ekiti Nigeria - Radiology, Ido Ekiti, Ekiti, Nigeria.; 2 Ladoke Akintola University of Technology College of Health Sciences - Radiology, Osogbo, Osun, Nigeria.; 3 Obafemi Awolowo University Teaching Hospitals Complex - Radiology, Ile-Ife, Osun, Nigeria.; 4 Ladoke Akintola University of Technology Teaching Hospital - Obstetrics and Gynecology, Osogbo, Osun, Nigeria.; 5 Faculdade de Medicina de Ribeirão Preto da Universidade de São Paulo (FMRPUSP), Ribeirão Preto, SP, Brazil.

**Keywords:** Hypertension, Proteinuria, Ultrasonography, Doppler, Vascular resistance, Placenta/physiology, Pregnancy

## Abstract

**Objective:**

To evaluate the resistive indices (RIs) of the fetal umbilical and middle cerebral arteries, as well as to determine the cerebroplacental ratio (CPR), in fetuses of women with hypertension.

**Materials and Methods:**

This was a comparative cross-sectional study involving 75 pregnant women with pregnancy-induced hypertension (PIH) and 75 apparently healthy pregnant women (control group), all of whom were submitted to Doppler ultrasound examination of the fetal middle cerebral and umbilical arteries between 20 and 40 weeks of gestation. The two groups were compared in terms of the RI of the middle cerebral and umbilical arteries, as well as the CPR. The level of statistical significance was set at *p* ≤ 0.05.

**Results:**

The mean age was 32.4 ± 4.6 years in the PIH group and 32.6 ± 4.6 years in the control group (*p* = 0.633). The mean umbilical artery RI was significantly higher in the PIH group than in the control group (0.67 ± 0.14 vs. 0.61 ± 0.08; *p* = 0.012), whereas the mean middle cerebral artery RI was significantly higher in the control group (0.80 ± 0.05 vs. 0.76 ± 0.08; *p* = 0.001). Among the women in the PIH group, the mean CPR was significantly lower for those with proteinuria than for those without (1.07 ± 0.26 vs. 1.27 ± 0.22; *p* = 0.001).

**Conclusion:**

Maternal hypertension during pregnancy appears to be associated with increased fetal umbilical artery RI and reduced fetal middle cerebral artery RI, as well as with a low CPR. In pregnant women, the combination of PIH and proteinuria is also apparently associated with an increased risk of a low CPR.

## INTRODUCTION

Maternal hypertension during pregnancy is defined as a systolic blood pressure ≥ 140 mmHg and a diastolic blood pressure ≥ 90 mmHg on at least two occasions, at least 6 h apart^**(1)**^. For 2017, the estimated maternal mortality rate worldwide was 211 per 100,000 live births. Sub-Saharan Africa and Southern Asia collectively accounted for approximately 86% of all estimated maternal deaths in 2017^**(2)**^. Eclampsia, which is a consequence of hypertension in pregnancy, is one of the five leading causes of maternal death in those regions^**(3)**^. Pregnancy-induced hypertension (PIH) is thought to be a result of abnormal cytotrophoblast invasion of the spiral arteries leading to uteroplacental insufficiency^**(4)**^. One of the fetal complications of maternal hypertension is impaired uteroplacental blood flow, which may result in intrauterine growth restriction. Impaired placental perfusion precedes clinical manifestations and can be detected promptly by Doppler ultrasound.

Assessment of the fetal circulation is of utmost importance in fetal monitoring and the clinical management of hypertension. Doppler ultrasound provides a means to monitor the maternal and fetal circulation, not only in normal pregnancies but also in high-risk pregnancies such as those of women with hypertension. Doppler ultrasound is a noninvasive method to study fetal hemodynamics and has therefore become a well-established method of assessing fetal well-being^**(5)**^. Doppler ultrasound of the fetal middle cerebral artery (MCA) provides information on the hemodynamic redistribution that accompanies uteroplacental insufficiency. The resistive index (RI) of the fetal umbilical artery (UA) reflects downstream placental vascular resistance, which correlates with the severity of uteroplacental insufficiency^**(6)**^.

The cerebroplacental ratio (CPR) is the ratio between the Doppler indices of the fetal MCA and those of the UA^**(7)**^. It is a good predictor of neonatal outcomes and can be used to identify fetuses at risk of morbidity and mortality^**(8)**^.

The objective of this study was to determine the effect that maternal hypertension has on the fetal MCA and UA by comparing pregnant women with and without PIH in terms of the fetal artery RIs. We also attempt to determine the effect that maternal hypertension has on the CPR.

## MATERIALS AND METHODS

This was a comparative cross-sectional study carried out in the radiology department of a tertiary hospital between July 2017 and June 2018. Women from 20 to 45 years of age with singleton pregnancies were included at 20-40 weeks of gestation. The subjects were divided into two groups: PIH, comprising pregnant women with newly diagnosed PIH who had yet to commence pharmacological treatment; and control, comprising apparently healthy pregnant women with no comorbidities associated with intrauterine growth restriction. Women with multiple pregnancies were excluded, as were those in whom there were structural malformations, chromosomal abnormalities, or oligohydramnios.

The local research ethics committee approved the study, and all participating subjects gave written informed consent. At enrollment, blood pressures were rechecked with the subjects in a sitting position. Urine dipstick tests were also performed in order to screen for proteinuria. Proteinuria was defined as urinary protein excretion of ≥ 0.3 g/L (≥ 1+ reading on the dipstick).

### Ultrasound technique

The women in the sample were evaluated with a diagnostic ultrasound system (DC-3; Mindray Bio-Medical Electronics Co., Ltd., Shenzhen, China) and a 3.5-5.0 MHz curvilinear probe. Each pregnant woman was placed in a semirecumbent position with a slight lateral tilt during scanning. That minimizes the risk of developing supine hypotension syndrome due to caval compression. An initial obstetric scan was performed in order to measure the fetal biometry, as well as to rule out multiple gestation, oligohydramnios, polyhydramnios, and congenital malformation.

Prior to the assessment of the fetal MCA, the skull was scanned in order to determine the biparietal diameter. A color Doppler examination was then performed in a plane slightly closer to the base of the skull, where the MCA can be identified as a vessel coursing toward the probe from the circle of Willis, within the Sylvian fissure^**(9)**^. The range gate was placed within the medial third of the vessel, with an angle of insonation of less than 60°, occupying approximately one third of its internal diameter. Recordings were made when there was no fetal movement, because high amplitude fetal breathing movements could modify the flow velocity waveforms.

**Figure 1:**
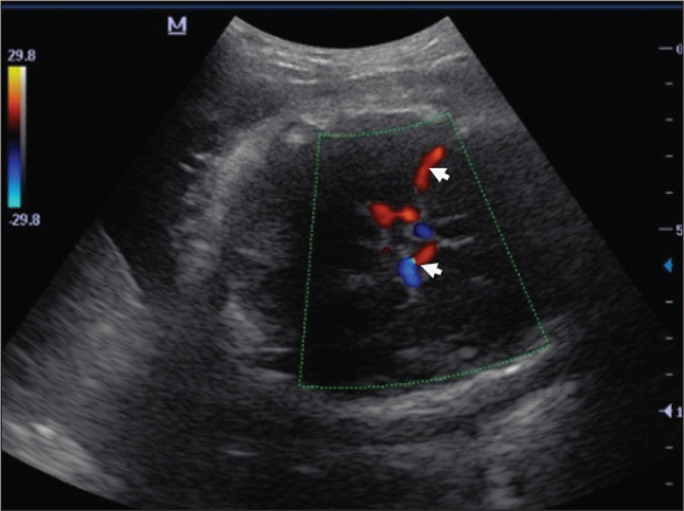
Color Doppler sonogram showing the fetal MCAs (arrows) in the Sylvian fissure.

**Figure 2:**
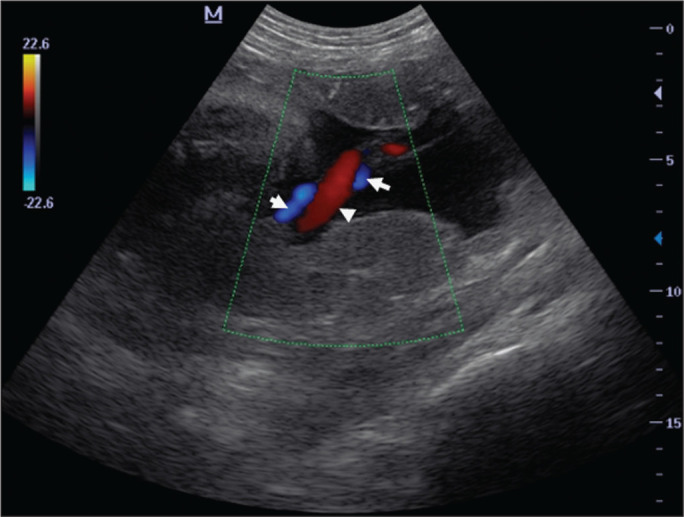
Color Doppler sonogram of the umbilical cord showing the UAs (arrows) and the umbilical vein (arrowhead).

**Figure 3:**
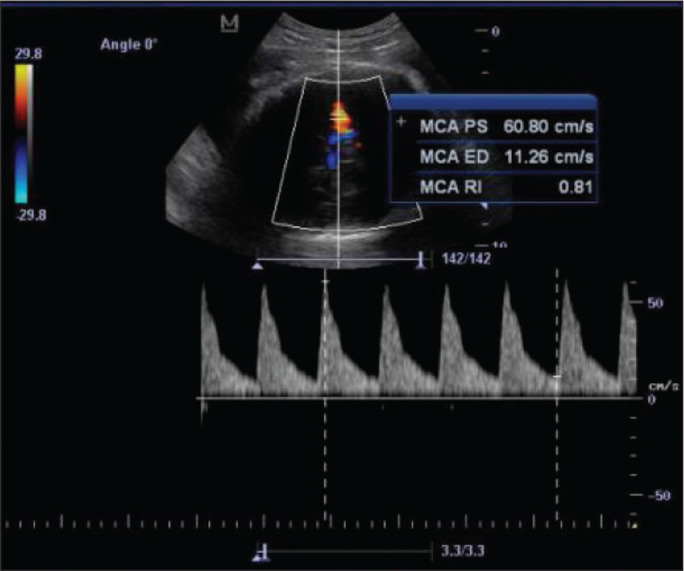
Color-flow sonogram of the MCA showing the typical biphasic flow.

**Figure 4:**
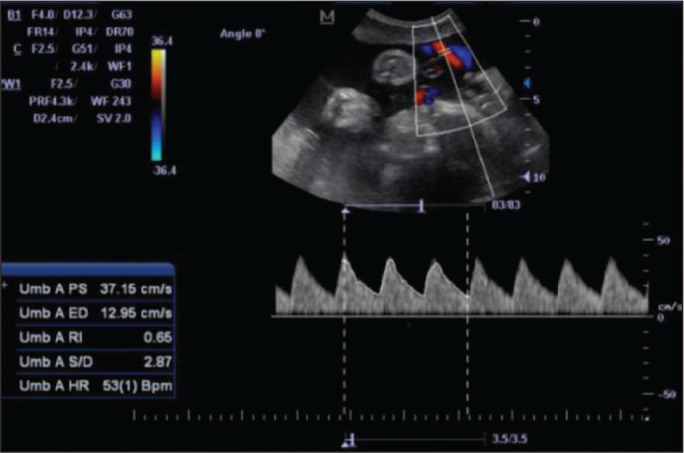
Color-flow sonogram of the UA showing the typical biphasic waveform with high diastolic flow at 33 weeks.

Either of the UAs was sampled at the middle of a free loop of the umbilical cord^**(9)**^, because the end diastolic flow is higher near the umbilical cord insertion into the fetal abdomen than near the placental insertion^**(10)**^. The CPR was calculated from the MCARI and UARI. For both vessels, measurements were taken three times by the same researcher and their means were determined in order to obtain a more accurate result and avoid intraobserver error (Figures 1 to 4).

### Statistical analysis

The MCARI, UARI, CPR, and other sonographic parameters of the pregnant women in both groups were recorded on the patient data sheet and entered into the computer spreadsheet using the IBM SPSS Statistics software package for Windows, version 20.0 (IBM Corp., Armonk, NY, USA). The data were analyzed using appropriate descriptive and inferential statistics. The results are expressed as mean ± standard deviation (SD) or as absolute and relative frequencies. The level of significance was set at *p* ≤ 0.05. Independent Student’s t-tests and analysis of variance F tests were used in order to compare the means of continuous variables between the two groups. Chi-square tests were used in order to evaluate associations between a CPR classified as ≤ 1 and that classified as > 1, as well as associations with the presence of proteinuria.

## RESULTS

A total of 150 pregnant women were recruited: 75 to the PIH group; and 75 to the control group. The general characteristics of the study population are shown in Table 1. Of the 75 women in the PIH group, 64 (85%) had no previous history of PIH, whereas 11 (15%) had had one or two previous episodes. The mean age was 32.6 ± 4.2 years in the control group and 32.4 ± 4.6 years in the PIH group (*p* = 0.633). The mean estimated gestational age was 31.6 ± 4.2 weeks in the control group and 31.1 ± 4.2 weeks in the PIH group (*p* = 0.453). The mean parity was 1.5 ± 0.9 for the women in the control group and 1.4 ± 1.2 for those in the PIH group (*p* = 0.065). There were statistically significant differences between the two groups in terms of the mean systolic and diastolic blood pressures, as well as the mean value for the mean arterial pressure, all of which were higher in the PIH group (*p* < 0.001 for all).

Table 2 shows the results of the urine dipstick test, which were normal (i.e., negative) for all of the women in the control group. Among the women in the PIH group, the result was negative in 47 (62.6%), 1+ in four (5.4%), 2+ in 12 (16.0%), and 3+ in 12 (16.0%). The differences between the two groups, in terms of the urine dipstick test results, were significant (*p* < 0.001).

**Table 1:** General characteristics of the pregnant women evaluated (N = 150).

Characteristic	Group		P
	PIH (n = 75)	Control (n = 75)	
Maternal age (years), mean ± SD	32.6 ± 4.2	32.4 ± 4.6	0.633
Gestational age (weeks), mean ± SD	31.1 ± 4.2	31.6 ± 4.2	0.453
Number of pregnancies, mean ± SD	1.4 ± 1.2	1.5 ± 0.9	0.065
History of PIH, n (%)	11 (15)	0 (0)	
Systolic blood pressure, mean ± SD	156.1 ± 16.3	110.6 ± 6.0	< 0.001
Diastolic blood pressure, mean ± SD	97.3 ± 8.9	75.1 ± 3.0	< 0.001

### Doppler indices

**Figure 5:**
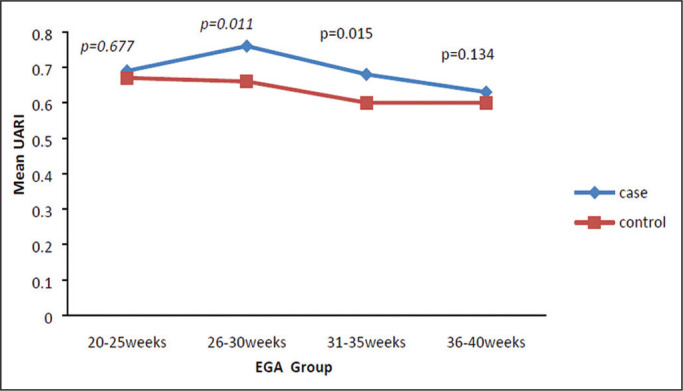
Line graph showing the mean UARI among the PIH and control group subjects, by gestational age group.

As shown in Table 3, the mean UARI was higher in the PIH group than in the control group (0.66 ± 0.10 vs. 0.63 ± 0.06) and the difference between the two groups was statistically significant (*p* = 0.028). As can be seen in Figure 5, the mean UARI among the women in the control group was highest at 20-25 weeks of gestation and lowest at 36-40 weeks. Across all of the gestational age ranges evaluated, the mean UARI was consistently higher in the PIH group, although the difference was statistically significant only for the 26-35 week range.

**Table 2:** Degree of proteinuria in the PIH and control group subjects.

Urine dipstick reading	Group		DF	χ^2^	P
	PIH n (%)	Control n (%)			
Trace (normal protein)	47 (62.7)	75 (100)	3	34.420	< 0.001
1+ (mild proteinuria)	4 (5.3)	0			
2+ (moderate proteinuria)	12 (16.0)	0			
3+ (severe proteinuria)	12 (16.0)	0			

**Table 3:** Mean UARI, MCARI, and CPR of fetuses in the PIH and control group subjects.

Variable	Group		F	P
	PIH Mean ± SD	Control Mean ± SD		
UARI	0.66 ± 0.10	0.63 ± 0.06	2.217	0.028
MCARI	0.76 ± 0.08	0.80 ± 0.05	3.365	0.001
CPR	1.178 ± 0.207	1.288 ± 0.156	9.670	0.001

**Figure 6:**
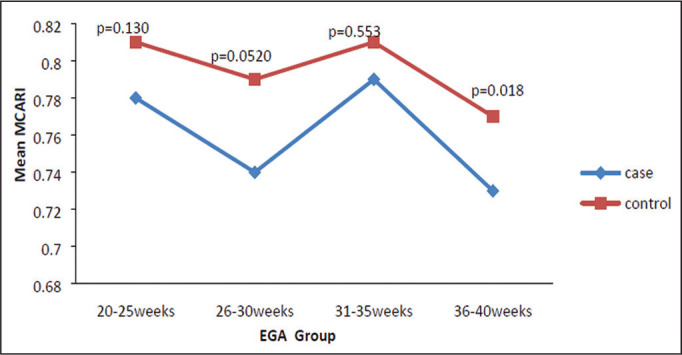
Line graph showing the mean MCARI among the PIH and control group subjects, by gestational age group.

The mean MCARI was lower in the PIH group than in the control group (Table 3), and the difference was statistically significant (*p* = 0.001). In both groups, the mean MCARI was lowest at 36-40 weeks of gestation and was lower in the PIH group than in the control group at all time points (Figure 6).

**Figure 7:**
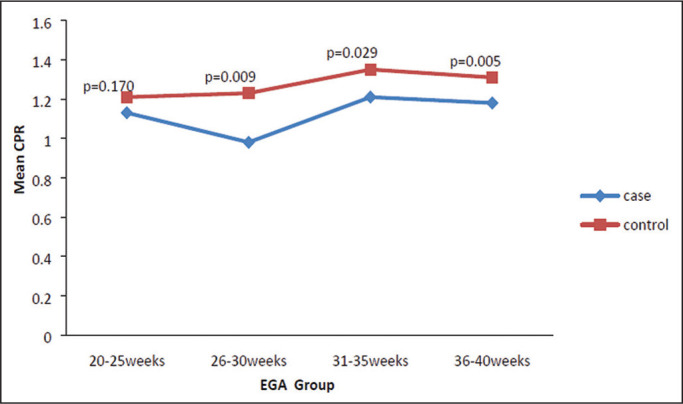
Line graph showing the mean CPR among the PIH and control group subjects, by gestational age group.

The mean CPR was lower in the PIH group than in the control group (Table 3), and the difference was statistically significant (*p* = 0.001). The mean CPR across the gestational age ranges are shown in Figure 7. The differences between the two groups became significant after 26 weeks of gestation (*p* = 0.170 at 20-25 weeks; *p* = 0.009 at 26-30 weeks; *p* = 0.029 at 31-35 weeks; and *p* = 0.005 at 36-40 weeks).

### Relationship of proteinuria with UARI, MCARI, and CPR

In the PIH group, the mean UARI was higher and the MCARI was lower among the subjects with proteinuria than among those without (Table 4). The difference was significant for the UARI but not for the MCARI. In addition, the CPR was lower among the PIH group subjects with proteinuria than among those without (1.07 ± 0.26 vs. 1.27 ± 0.22; *p* = 0.000).

**Table 4:** Mean MCARI, UARI, and CPR of fetuses among women with PIH, with and without proteinuria.

Variable	Proteinuria		t	P
	Yes (n = 28)	No (n = 47)		
UARI, mean ± SD	0.73 ± 0.01	0.62 ± 0.07	4.330	< 0.001
MCARI, mean ± SD	0.74 ±0.06	0.78 ± 0.06	1.896	0.062
CPR, mean ± SD	1.05 ± 0.02	1.25 ± 0.02	4.493	< 0.001

Among the 75 women in the PIH group, the CPR was = 1 in 16 (21.3%). Of the 47 PIH group subjects without proteinuria, 45 (95.7%) had a CPR > 1 and only 2 (4.3%) had a CPR ≤ 1, as shown in Table 5. Of the 28 PIH group subjects with proteinuria, 14 (50.0%) had a CPR ≤ 1 (Table 5). None of the control group subjects had a CPR ≤ 1.

**Table 5:** Chi-square analysis of the CPR of fetuses among women with PIH, with and without proteinuria.

Urinary protein excretion	CPR		DF	χ2	P
	≤ 1 n (%)	> 1 n (%)			
Normal	2 (12.5)	45 (76.3)	1	21.879	< 0.001
Proteinuria	14 (87.5)	14 (23.7)			

## DISCUSSION

In low- and middle-income countries, PIH is one of the leading causes of maternal morbidity and mortality^**(2,11)**^. The disease is caused by abnormal placentation leading to inadequate trophoblastic invasion of the maternal spiral arterioles, resulting in increased vascular resistance and reduced uteroplacental perfusion. Doppler ultrasound provides information on uteroplacental vascular resistance and, indirectly, on the fetal blood circulation^**(12)**^.

In our study, the UARI among the control group subjects decreased gradually over the course of the pregnancy, as was expected. This is in keeping with the results of some previous studies. In two different longitudinal studies, conducted in Switzerland and Norway, respectively, Kurmanavicius et al.^**(13)**^ and Acharya et al.^**(14)**^ both reported that the UARI decreased with advancing gestational age. In a similar study, conducted in Nigeria, Ayoola et al.^**(15)**^ reported that the mean UARI declined between 15 and 39 weeks of gestation. In a normal pregnancy, as the placenta develops, there is a normal drop in vascular resistance in the UA as a result of increases in the numbers of stem villi and small arterial vessels. That causes a gradual decline in the UARI as gestational age increases^**(10)**^. However, such a decrease does not occur during the pregnancies of women with hypertension. When there is placental insufficiency, as in cases of PIH, there is a decrease in the number of capacitance vessels, resulting in high resistance (i.e., an increased RI) in the UA^**(16)**^. That could explain why the range of mean UARI values in our PIH group was comparable across the various gestational age ranges. Our finding that the mean UARI was higher in the PIH group that in the control group is in agreement with the findings of Gupta et al.^**(17)**^, who reported that, among women with PIH in India, all three of the Doppler indices measured (the pulsatility index, the RI, and the systolic/diastolic ratio) declined as in a normal pregnancy, although the individual values were usually found to be > 2 standard deviations above the mean for the same gestational age in controls^**(17)**^. In a similar study, conducted in Mexico, Lopez-Mendez et al.^**(18)**^ reported that the mean UARI was higher in the fetuses of pregnant women with preeclampsia than in those of normotensive pregnant women. In another study conducted in Nigeria, Udo et al.^**(19)**^, also reported that the UARI was higher in the fetuses of pregnant women with hypertension, as were other parameters. In a similar study, conducted in India, Lakhar et al.^**(20)**^ found that the RI values were lower in the fetuses of women with normal pregnancies than in those of same gestational age of pregnant women with hypertension.

In the present study, the difference between the PIH and control groups, in terms of the mean UARI, was found to be more statistically significant between 26 and 35 weeks of gestation. That was an expected finding, because early-onset PIH (i.e., that occurring before 34 weeks of gestation) is associated with greater maternal and fetal morbidity and mortality than is the late-onset type^**(21)**^.

In our sample, the mean MCARI was 0.76 in the PIH group, whereas it was 0.80 in the control group. Those values are similar to the 0.76 and 0.83, respectively, reported by Lopez-Mendez et al.^**(18)**^. In another study, conducted in Turkey, Ozerem et al.^**(22)**^ assessed resistance in the fetal MCA by measuring the pulsatility index, which they found to be lower in the fetuses of pregnant women with preeclampsia than in those of women with normal pregnancies, suggesting that fetal cerebral blood flow resistance is lower in the former.

Changes in the fetal cerebral circulation are predictive of the condition of the fetus. The MCA is the vessel of choice to assess the fetal cerebral circulation^**(5)**^. In a normal pregnancy, the RI of the fetal MCA declines gradually as gestation progresses^**(23)**^. That is because the fetal MCA is a low-resistance vessel throughout pregnancy. In two separate longitudinal cross-sectional studies, the previously cited study conducted by Kurmanavicius et al.^**(13)**^ and the study conducted in Iran by Tarzamni et al.^**(5)**^, the MCARI followed a parabolic pattern, with a peak at week 28 of gestation. However, in our study, the control group MCARI values did not follow any particular pattern, although they were consistently higher than the PIH group values for fetuses of the same gestational age. That discrepancy could be due to differences in the ethnicity of the study populations or to the larger sample sizes in those two studies (1,675 and 1,037, respectively, vs. 150 in the present study). In our study, the mean MCARI in the PIH and control groups was lowest at 36-40 weeks of gestation (0.73 and 0.77, respectively). Abnormal Doppler indices for the MCA have been associated with intrauterine growth restriction, preeclampsia, and fetal hypoxia^**(24)**^. When there is fetal hypoxemia, as may occur in uteroplacental insufficiency, chemoreceptors are stimulated, triggering a response that leads to vasodilatation of vital organs, including the brain.^**(24)**^ There is a relationship between vasodilatation of the MCA and fetal hypoxemia^**(23)**^. Such vasodilatation causes a drop in the resistance of the fetal cerebral circulation, which further decreases the MCARI. When hypoxemia and acidemia become more severe, the RI reaches a nadir, which purportedly represents the maximum vessel dilatation, after which the fetal heart rate starts to decelerate^**(25)**^.

The CPR, as used in the present study, is the ratio between the fetal MCARI and the fetal UARI. The CPR describes the correlation of placental resistance with cerebral adaptation to placental insufficiency^**(9)**^. The ratio is considered abnormal when less than one^**(26)**^. Various studies have shown that the CPR is a useful tool for predicting fetal outcomes in pregnancies complicated by maternal hypertension^**(26-28)**^. In the present study, 16 (21.3%) of the 75 PIH group subjects had a CPR ≤ 1. None of the control group subjects had a CPR ≤ 1. In a study carried out in Kenya, Parshuram et al.^**(26)**^ found a CPR ≤ 1 in 47 (29.4%) of 160 cases of PIH. Their slightly larger sample size could account for the fact that the proportion of fetuses with an abnormal CPR was higher in their study than in ours. Those authors also found that the numbers of stillbirths and neonates with a birth weight below the 10th percentile were highest among the subgroup with a fetal CPR ≤ 1. In a similar study, conducted in Turkey and involving 50 pregnant women with PIH, Yalti et al.^**(28)**^ found a CPR ≤ 1 in 16 (32.0%) of the cases, all of which had worse fetal outcomes.

In the present study, 14 (87.5%) of the 16 subjects with a CPR ≤ 1 had proteinuria, even though the prevalence of proteinuria in the PIH group was only 37.3%. Similarly, the mean UARI was higher and the mean MCARI was lower in the PIH group subjects with proteinuria than in those without. These findings suggest that the presence of proteinuria is associated with worse fetal complications in the setting of maternal hypertension. Other studies have shown proteinuria to be associated with greater disease severity and worse fetal outcomes^**(29)**^. However, to our knowledge, there have been no previous studies demonstrating the relationship between proteinuria and the severity of changes in the fetal Doppler indices in PIH. In the present study, we have shown that the effect of PIH on the fetal Doppler indices is compounded by the presence of proteinuria, which may further worsen perinatal outcomes.

## CONCLUSION

Maternal hypertension during pregnancy appears to be associated with increased fetal UARI and reduced fetal MCARI, as well as with a low CPR. In pregnant women, the combination of PIH and proteinuria is also apparently associated with an increased risk of a low CPR. Therefore, pregnant women with hypertension should be monitored by Doppler ultrasound throughout gestation and should be screened for proteinuria on a regular basis.
